# Inequalities in the use of public oral health services among older adults in Brazil: a national population-based study, 2023

**DOI:** 10.1590/1980-549720260004.supl.1

**Published:** 2026-04-17

**Authors:** Breno Marques de Mello, Debora Lana Alves Monteiro, Ayla Miranda de Oliveira, Bárbara Rachelli Farias Teixeira, Raissa Taynnar Albuquerque Lopes, Rênnis Oliveira da Silva, Yuri Wanderley Cavalcanti, Rafael Aiello Bomfim

**Affiliations:** I Universidade Federal da Paraíba, Graduate Program in Odontology – João Pessoa (PB), Brazil.; II Universidade Federal da Paraíba, João Pessoa Health Sciences Center, Department of Clinical and Social Odontology – João Pessoa (PB), Brazil.; III Universidade Federal de Mato Grosso do Sul, Dental School, Department of Social Odontology – Campo Grande (MS), Brazil.

**Keywords:** Oral health, Social inequalities, Elderly, Public health services

## Abstract

**Objective:**

To analyze individual and contextual factors associated with the use of public dental services among Brazilian older adults, using data from SB Brasil 2023.

**Methods:**

A total of 9,745 older adults were included in this cross-sectional study. The outcome was the use of public oral health services versus private services. Analyses accounted for the complex sampling design and applied multilevel logistic regression with a random intercept at the municipal level, guided by Andersen's Behavioral Model. Independent variables included predisposing factors (race/skin color, sex, and level of education), enabling factors (per capita income and oral health coverage in Primary Health Care) and need factors (edentulism and perceived need for dental treatment and prostheses).

**Results:**

The weighted prevalence of public dental service use was 39.2% (95%CI 35.7–42.9). After adjustment, higher odds of using the public system were observed among Black (OR 1.97; 95%CI 1.60–2.40), mixed-race (OR 1.38; 95%CI 1.19–1.61), and Indigenous older adults (OR 5.16; 95%CI 2.01–13.21), as well as among those with edentulism (OR 1.44), perceived need for dental treatment (OR 1.26), and need for prostheses (OR 1.39). Living in municipalities with oral health coverage above 70% was associated with greater use of public services (OR 2.47; 95%CI 1.83–3.31). The proportion of variance attributable to the municipal level decreased from 28.3% in the null model to 16.7% in the final model.

**Conclusion:**

Public dental service use among older adults in Brazil is socially stratified and strongly associated with social vulnerability and oral health needs, highlighting the pro-equity role of the Brazilian Unified Health System.

## INTRODUCTION

The use of public oral health services in Brazil is permeated by important social, racial, gender, and territorial inequalities^1^. The older adult population, a priority group regarding the need for oral rehabilitation, experiences these asymmetries within a context of greater economic and social vulnerability^2,3^. There is evidence that factors — such as lower income, low level of education, and edentulism — are associated with greater use of public oral health services, reflecting not only patterns of inequality in access, but also the strategic role of the Brazilian Unified Health System (SUS) in expanding coverage and in the potential to mitigate inequalities in oral health^4,5^.

The Andersen's Behavioral Model is a widely consolidated theoretical framework for the analysis of the use of health services, postulating that the use of services results from the interaction between predisposing, enabling, and need factors, operating at individual and contextual levels. In this study, individual predisposing factors comprised sociodemographic characteristics such as age, sex, race/skin color, and level of education. The enabling factors included individual attributes, especially per capita income, as well as contextual characteristics of the municipality of residence, such as oral health coverage in Primary Health Care (PHC) and municipal racial composition. The need factors were evaluated exclusively at the individual level, considering both clinical and perceived needs, including edentulism, self-perception of the need for dental treatment, self-perception of the need for prosthesis, and reason for using the service (treatment, prevention, or prosthesis placement/maintenance). This analytical structure allows us to systematically investigate how individual and contextual determinants articulate to shape patterns of use of public oral health services in a universal health system^6^.

In the Brazilian context, it is essential to incorporate the municipal dimension in the analysis of the use of oral health services, considering the principle of territorialization of PHC and its structuring role in the organization of care. The distribution and local coverage of PHC not only condition the supply and access to dental services, but also interact with social markers — such as race/skin color, income, age, and sex —, producing differentiated patterns of use among population groups^1,7,8^. This perspective is particularly relevant in a universal system, in which equity in access must be understood as a result of the articulation between individual characteristics and the organizational capacity of services at the local level.

According to the literature, equity in access to and use of public health services is not restricted to users’ individual attributes, being intrinsically related to the way health systems are organized, funded, and implemented in the territories^8^. Nevertheless, studies whose authors adopt multilevel approaches to analyze the use of dental services by older adults, simultaneously incorporating contextual variables of the territory, the municipal demographic and racial composition, and indicators of need in oral health, are still scarce^9,10^. Moreover, even among investigations using multilevel models, it is observed that most do not systematically explore variables related to race/skin color, self-perception of the need for prosthesis, and reasons for using the services, limiting the understanding of intersectional inequalities in access to dental care^11^.

Given this scenario, in the present study, we aimed to analyze the individual and contextual factors associated with the use of public oral health services among Brazilian older adults, based on data from the National Survey of Oral Health — SB Brasil 2023, in light of Andersen's Behavioral Model, contributing to the debate on equity, territorial organization of PHC, and reduction of inequalities in oral health within the SUS.

## METHODS

This is a cross-sectional population-based study with data from the national survey SB Brasil 2023 — National Survey of Oral Health, coordinated by the Ministry of Health. The research was conducted between 2022 and 2023 and followed standardized methodological guidelines throughout the national territory, and the study design was described by Vargas et al.^12^ and Ferreira et al.^13^

The sampling design was probabilistic and stratified, ensuring representativeness for the capitals of the 26 states, the Federal District, and urban areas of small cities. The selection of the sample was carried out in different stages, with previous listing and systematic draw of households, in addition to the use of electronic devices and specific systems of data collection and validation in real time.

The collection included household interviews and standardized clinical examinations, comprising demographic, socioeconomic variables, self-perception of oral health, self-reported need for treatment, and use of dental services. Data were collected by dentists and annotators previously trained and calibrated using standardized instruments such as structured questionnaires and oral clinical examinations. Data standardization and quality were ensured through pilot tests, regular oversight, and electronic collection system with real-time validation^12,13^.

In the present study, 9,745 older adults aged 60 years or over, who participated in the survey, and who presented complete information for the variables of interest were analyzed. The main outcome was the use of public oral health services, identified based on the question about the location of the last dental consultation. The answers were dichotomized between care provided by the SUS and other types of service (private, health insurance, or others).

To analyze factors associated with the use of public oral health services in the SUS, the analyses were guided by the behavioral model of Andersen and Newman^6^, according to which the use of health services results from the interaction between predisposing, enabling, and need factors, operating at individual and contextual levels. Considering the hierarchical structure of the data of SB Brasil 2023, with individuals clustered in municipalities, multilevel logistic regression with random intercept per municipality was used, allowing to simultaneously estimate the effects of individual and contextual variables as well as the variance attributable to the municipal level.

Independent variables were organized according to the components of the theoretical model. Individual predisposing factors included sex, self-reported race/skin color, and level of education. Enabling factors included individual attributes, such as per capita income, and contextual characteristics of the municipality of residence, including oral health coverage in PHC, and proportion of the Black population in the municipality. Need factors were evaluated exclusively at the individual level and comprised both clinical and perceived needs, including edentulism, self-perception of the need for dental treatment, self-perception of the need for dental prosthesis, evaluation of oral health, and reason for using the service.

Sequential hierarchical models were adjusted, starting with a null model, followed by models with progressive inclusion of individual and contextual variables, according to Andersen's theoretical framework. Analyses were performed using the Stata software, version 14.2 (StataCorp, USA, TX), considering the sample weights and the effect of the complex sampling design of SB Brasil 2023. The results were expressed as odds ratios (OR) and 95% confidence intervals (95%CI).

**Figure 1:**
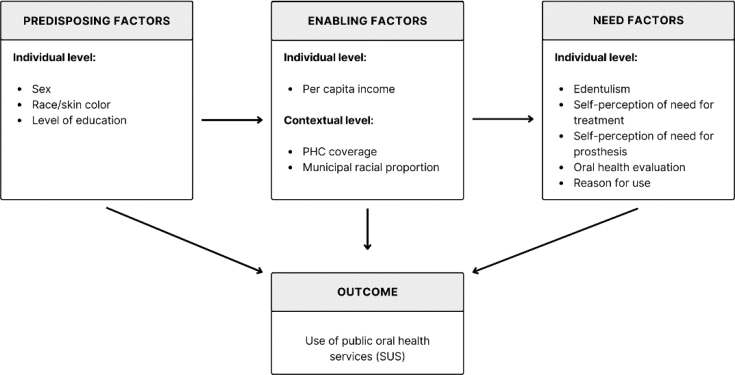
Conceptual framework of Andersen's Behavioral Model for the use of public oral health services in Brazil (older adults).

In Figure 1, the Andersen's conceptual model is illustrated to explain the use of public oral health services by older adults.

The SB Brasil 2023 project was conducted in accordance with Resolution No. 466/2012 of the National Health Council and was approved by the National Commission of Ethics in Research (*Comissão Nacional de Ética em Pesquisa* – CONEP) under Certificate of Presentation for Ethical Consideration (CAAE) No. 34497120.6.3001.0008. All participants or their legal guardians signed the Informed Consent Form and, for children under 18 years of age, the Informed Assent Form was also obtained.

### Data availability statement

The entire dataset that supports the results of this study is available upon request to the author.

## RESULTS

The study included 9,745 older adults who participated in the National Survey of Oral Health — SB Brasil 2023. In Table 1, we present the descriptive characteristics of the studied population, while in Table 2 we present the results of the multilevel logistic regression, organized according to the components of Andersen's behavioral model.

**Table 1:** Descriptive characteristics and proportions. SB Brasil 2023 Study (n=9,745).

Individual variables			n	% (weighted proportion)	Public use
					% (95%CI)
Prevalence					39.2% (35.7–42.9)
Ethnic group					
	White		3,601	49.5 (45.8–53.3)	32.0 (27.7–36.7)
	Mixed-race		4,465	36.1 (32.4–40.2)	44.5 (38.5–50.5)
	Asian		129	1.1 (0.7–1.7)	39.7 (25.3–56.1)
	Black		1,353	12.8 (11.2–14.7)	52.9 (46.2–59.4)
	Indigenous		50	0.4 (0.2–1.0)	76.4 (51.2–90.9)
	Ignored/No information		147	1.2 (0.7–2.0)	37.0 (19.3–59.1)
Sex					
	Women		6,034	60.4 (58.2–62.7)	39.1 (35.7–42.7)
	Men		3,710	39.6 (37.3–41.9)	39.4 (33.8–45.2)
Per capita income (minimum wage)					
	Up to the poverty line		621	6.7 (5.3–8.4)	56.6 (45.2–67.4)
	Poverty line to 1		2,263	27.6 (24.5–30.9)	51.9 (45.2–58.6)
	From 1 to 2		2,891	44.4 (40.7–48.3)	36.5 (31.1–42.3)
	Above 2		1,409	21.2 (18.0–24.9)	20.5 (13.2–30.5)
	Ignored/No information		2,561	27.7 (23.4–32.5)	41.9 (35.2–48.8)
Level of education (years)					
	Illiterate		1,226	11.4 (9.8–13.3)	63.4 (55.8–70.4)
	From 1 to 4		2,485	58.7 (55.4–61.9)	42.3 (37.2–47.6)
	From 5 to 8		2,301	20.7 (18.2–23.5)	30.4 (25.1–36.2)
	Above 8		998	9.2 (7.4–11.3)	12.0 (8.4–16.9)
	Ignored/No information		235	3.8 (2.4–5.7)	50.6 (37.6–63.5)
Contextual					
	PHC oral health coverage				
		Up to 70%	7,300	72.3 (67.0–77.0)	34.0 (29.8–38.6)
		>70%	2,445	27.7 (22.9–33.0)	53.1 (47.6–58.5)
Municipal racial proportion					
	Up to 60% Black individuals		4,494	65.8 (61.4–70.0)	33.0 (28.5–37.9)
	>60% of Black individuals		5,291	34.2 (30.0–38.6)	51.9 (47.3–56.4)
Edentulous					
	No		6,230	63.5 (60.4–66.6)	38.1 (32.7–42.7)
	Yes		3,490	36.5 (33.4–39.6)	41.3 (36.6–46.2)
Self-perception of the need for dental treatment					
	No		4,434	49.0 (45.7–52.2)	32.1 (28.1–36.4)
	Yes		5,038	51.0 (47.8–54.3)	46.1 (41.6–50.6)
Self-perception of the need for prosthesis					
	No		3,793	42.6 (39.8–45.5)	33.5 (29.1–38.2)
	Yes		5,543	57.4 (54.5–60.2)	42.3 (37.3–47.5)
Reason					
	Treatment		4,092	45.5 (42.2–48.9)	46.9 (41.5–52.4)
	Prevention		1,396	17.1 (14.4–20.2)	38.1 (29.8–47.2)
	Prosthesis placement/maintenance		2,955	37.4 (22.9–41.1)	28.8 (24.1–34.1)

95%CI: 95% confidence interval.

**Table 2:** Factors associated with the use of public dental services (multilevel logistic regression).

Variables			Use of public services (OR)	
			Not adjusted	Adjusted*
			OR (95%CI)	OR (95%CI)
Predisposing individual factors				
Race/skin color				
	White		1	1
	Black		2.18 (0.84–1.29)	1.97 (1.60–2.40)
	Asian		1.47 (0.98–2.20)	1.53 (0.84–2.81)
	Mixed-race		1.66 (1.48–1.85)	1.38 (1.19–1.61)
	Indigenous		4.79 (2.36–9.72)	5.16 (2.01–13.21)
Sex				
	Men		1	1
	Women		0.94 (0.83–1.02)	–
Level of education (years of study)				
	0		1	1
	1 to 4		0.66 (0.55–0.78)	0.65 (0.51–0.81)
	5 to 8		0.63 (0.53–0.75)	0.72 (0.58–0.91)
	>8		0.30 (0.26–0.36)	0.37 (0.30–0.47)
Individual enabling factors				
Per capita income (equivalent) (minimum wage)				
	Up to the poverty line		1	1
	Poverty line to 1		0.71 (0.57–0.88)	0.84 (0.66–1.07)
	1 to 2		0.50 (0.41–0.62)	0.67 (0.53–0.86)
	Above 2		0.16 (0.12–0.20)	0.26 (0.20–0.35)
Contextual enabling factors				
PHC oral health coverage				
	Up to 70%		1	1
	Above 70%		2.83 (2.13–3.74)	2.47 (1.83–3.31)
Racial proportion				
	Up to 60% Black individuals			
	Above 60% Black individuals		2.38 (1.77–3.19)	1.25 (0.93–1.69)
Individual need factors				
Edentulism				
	No			
	Yes		0.84 (0.75–0.92)	1.44 (1.22–1.70)
Need for dental treatment				
	No			
	Yes		1.80 (1.63–1.99)	1.26 (1.08–1.46)
Self-assessment of need for prosthesis				
	No			
	Yes		1.75 (1.59–1.93)	1.39 (1.21–1.59)
Reason for consultation				
	Prevention		1	
	Treatment		1.68 (1.46–1.92)	1.09 (0.92–1.30)
Prosthesis maintenance/placement			0.38 (0.33–0.44)	0.19 (0.16–0.24)
	AIC	null model	11,198	6,575
	BIC	null model	11,212	6,702
	%CV	null model	28.3% (22.7–34.7)	16.7% (11.4–23.7)

OR: odds ratio; 95%CI: 95% confidence interval; AIC: Akaike Information Criterion; BIC: Bayesian Information Criterion; %CV: Percent Coefficient of Variation (% of variation at the municipal level).

### Individual predisposing factors

In the block of predisposing factors, race/skin color maintained a significant association with the use of public dental services after adjustment by individual and contextual variables. We verified higher odds of using the SUS among Black individuals (OR 1.97; 95%CI 1.60–2.40), mixed-race (OR 1.38; 95%CI 1.19–1.61), and Indigenous people (OR 5.16; 95%CI 2.01–13.21) when compared to white older adults. We found no statistically significant association in the adjusted model for the Asian category.

Sex was not independently associated with the use of public dental services. Regarding level of education, we observed an inverse gradient: the higher the level of education, the lower the odds of using SUS. We verified a significant reduction in the odds of use (OR 0.37; 95%CI 0.30–0.47) of older adults with over eight years of formal study when compared to illiterate people.

### Individual and contextual enabling factors

Among the individual enabling factors, there was an inverse association between per capita income and the use of public services. Older adults with income between one and two minimum wages had lower odds of using the SUS (OR 0.67; 95%CI 0.53–0.86), and those with income above two minimum wages presented 74% lower odds of using it (OR 0.26; 95%CI 0.20–0.35) in relation to poor older adults.

At the contextual level, we found a robust association between oral health coverage in PHC and the outcome. Older adults living in municipalities with coverage greater than 70% had more than twice the odds of using public dental services (OR 2.47; 95%CI 1.83–3.31) compared to those living in municipalities with lower coverage. Conversely, the municipal racial proportion did not maintain a statistically significant association after adjustment (OR 1.25; 95%CI 0.93–1.69).

### Individual need factors

In the block of need factors, we verified a positive association between edentulism and the use of public dental services in the adjusted model (OR 1.44; 95%CI 1.22–1.70). Likewise, older people who reported need for dental treatment (OR 1.26; 95%CI 1.08–1.46) and need for dental prosthesis (OR 1.39; 95%CI 1.21–1.59) presented higher odds of using the SUS.

As for the reason for consultation, the search for prosthesis maintenance or placement was associated with lower odds of using public services (OR 0.19; 95%CI 0.16–0.24), when compared to consultations due to preventive reasons. The reason for treatment did not maintain a statistically significant association after adjustment.

#### Variance between municipalities

According to the multilevel analysis, in the null model, 28.3% of the variability in the use of public dental services was attributable to differences between the municipalities. After including the individual and contextual variables, this percentage decreased to 16.7%, demonstrating that a substantial part of the variation in the use of the SUS is explained by factors contemplated by the Andersen's behavioral model.

## DISCUSSION

In the present study, based on data from SB Brasil 2023, we analyzed the use of public dental services in the light of Andersen's behavioral model, according to which the use of services results from the interaction between predisposing, enabling, and need factors, operating at individual and contextual levels. In the component of predisposing factors, we observed a higher probability of SUS use among self-reported Black, mixed-race, and Indigenous older adults, regardless of other individual and contextual characteristics. Within the scope of the enabling factors, lower per capita income and lower levels of education were highlighted as central determinants of greater dependence on public dental services, evidencing an inverse socioeconomic gradient in the use of the SUS. In turn, the need factors, represented by edentulism and the perception of the need for treatment or dental prosthesis, were strongly associated with the use of services, showing that the SUS acts primarily in meeting the accumulated and perceived demands in oral health among older people with greater social and clinical vulnerability.

In addition, we observed a lower probability of use of public services among older people who sought care for preventive reasons (such as routine consultations and educational actions) or rehabilitation reasons (for instance, dental prostheses manufacture and maintenance), when compared to those who sought services due to the need for curative treatment such as extractions, restorations, or pain management. At the contextual level, municipalities with higher population coverage by oral health teams (OHT) in PHC and higher proportion of Black population showed higher odds of using public services.

In a scoping review, Ghanbari-Jahromi et al.^14^ identified several factors that influence the use of dental services among older adults, highlighting income, level of education, perception of need, edentulism, and contextual barriers such as availability and cost. These findings are consistent with the results of the present study, as we also highlighted that socioeconomic factors and perception of need are related to the preferential use of the SUS.

Andersen's behavioral model, proposed in 1968 and reviewed over the decades^15^, is one of the theoretical models applied to understand the use of health services. According to this model, the use of services is influenced by three dimensions: predisposing factors (such as age, level of education, and race/skin color), enabling factors (such as income and availability of services), and need factors (such as perception of health status or presence of clinical conditions). In this study, we identified higher odds of using public services among older people who sought care for curative reasons, which reinforces the relevance of need factors as the main reason for seeking dental care. Moreover, we verified that municipalities with higher coverage by OHT presented higher odds of using these services, indicating the OHT presence as an enabling factor related to network availability. The higher prevalence of use of public services among Black people can be understood as a predisposing factor in the Andersen's model, considering that this population historically has greater dependence on the SUS. In a study, Constante identified that mixed-race and Black individuals are, respectively, 1.25 and 1.73 times more likely to use public dental services compared to whites^16^.

The findings of this study corroborate Martins et al.^17^, who, when analyzing data from SB Brasil 2010, identified greater use of public services by older adults with low income, lower levels of education, Black or mixed-race, and with negative self-perception of oral health. The presence of OHT in the municipality was also associated with the use, highlighting the influence of enabling and contextual factors in the expansion of access. Our findings follow the pattern found in SB 2010.

Furthermore, Moreira et al.^18^, by analyzing latent classes, also stressed that the "SUS profile" group included older people with worse socioeconomic conditions, need for prosthesis, and negative perception of oral health, characteristics similar to those found in our study. It should be noted that, over 13 years, the profile of people who access the SUS did not significantly change. This somewhat evidences that the public health sector welcomes the people who need it most. Conversely, this finding is alarming, given the amount invested in the oral health of the SUS. Considering the older adult population, we can infer that improvements resulting from the implementation and expansion of oral health in the SUS were not sufficient to produce changes in the oral condition of these individuals, especially due to the accumulation of oral problems at a time prior to the National Oral Health Policy (*Política Nacional de Saúde Bucal* – PNSB).

As per the analysis conducted by Pinto et al.^19^, most recent data from SB Brasil 2023 corroborate this pattern, with greater use of the SUS among adults aged 35 to 44 years with low levels of education, low income, Black or mixed-race, without health insurance, with total edentulism, worse self-perception of oral health, and perceived need for treatment. Although the study focused on adults, the observed patterns coincide with those found among older individuals in this study, emphasizing the persistence of inequalities in access. Even though PNSB has expanded access to dental services within the SUS and contributed to the reduction of racial and social inequalities in oral health^20^, according to our findings, the use of public services remains concentrated among more socially vulnerable groups. Far from representing a limitation of the SUS, this pattern reinforces its role as a pro-equity mechanism, by ensuring priority access to those with a higher burden of accumulated needs in oral health. The persistence of this profile of users over time should be interpreted in the light of historical structural inequities, marked by late or insufficient access to dental services throughout the course of life, especially among the older adult population. Hence, the high demand observed among the most vulnerable older adults reflects, to a large extent, the accumulation of oral health problems prior to the implementation of the PNSB, as well as the chronic and cumulative nature of oral health conditions, and not an insufficiency of current public policies to modify, in the short term, outcomes strongly determined by past social trajectories.

Although the findings suggest greater use of public services among edentulous older adults, the regression indicated lower odds of use, suggesting that edentulism can act as a barrier to access, especially among the most vulnerable. Total or partial teeth loss, associated with other inequalities, can lead to obstacles that make the use of public oral health services impossible. One possible explanation is the naturalization of tooth loss as part of aging, especially among older people with lower levels of education. In the absence of evident pain or functional discomfort, the search for treatment, such as prosthetic rehabilitation, tends to be neglected. This behavior can be interpreted in the light of the Andersen's model^21^, according to which the subjective perception of need is deemed a central determinant of the use of services.

Authors of studies with data from SB Brasil 2010 identified a high need for prosthesis among older people with low levels of education, income, and worse self-rated health, highlighting inequalities in access to rehabilitation^22,23^. Ribeiro et al.^24^ state that the need for prosthesis does not always result in search for care due to the combination of individual and contextual factors such as insufficient income, low level of education, reduced mobility, and insufficient public services. This scenario suggests that, for many older adults, prosthetic rehabilitation is no longer a priority in the face of other daily limitations. In addition, there are indications of greater search for rehabilitation in private services, possibly due to greater agility or perceived quality. Nascimento et al. observed that older adult users of total prosthesis had higher odds of care in private services^25^.

Fagundes et al., based on data from PNS 2019, showed greater use of dental services among people with higher income and level of education, indicating a predominance of private access, while among older adults the prevalence was lower, demonstrating persistent barriers for more vulnerable groups^26^. On the international scene, Cossio-Alva et al. identified that the perception of need was the main factor associated with the use of services among Peruvian older adults, reinforcing the role of social determinants^27^.

In this study, we found a strong association between the need factors and the use of public services, in interaction with predisposing and enabling factors. The lower odds of use among edentulous older adults, even in the face of the need, reinforces the existence of barriers to rehabilitating care. Despite the limitations inherent in the cross-sectional design and the use of self-reported variables, the findings are relevant to guide public policies aimed at expanding rehabilitating access and denaturalization of edentulism in old age. The use of multilevel logistic regression allowed us to consider individual and contextual factors, increasing the understanding of the determinants of public dental services use by older adults.
