# Supplementary material for: Inequalities in the use of public oral health services among older adults in Brazil: a national population-based study, 2023
Source: Rev Bras Epidemiol. 2026 Apr 17;29(Suppl 1):e260004supl1. doi: 10.1590/1980-549720260004.supl.1 (PMC13089318; doi:10.1590/1980-549720260004.supl.1)
Supplement: Translation

# Translation: Desigualdades no uso de serviços públicos de saúde bucal por idosos no Brasil: estudo nacional de base populacional, 2023

**DOI:** 10.1590/1980-549720260004.supl.1.2

**Authors:** Breno Marques de Mello, Debora Lana Alves Monteiro, Ayla Miranda de Oliveira, Bárbara Rachelli Farias Teixeira, Raissa Taynnar Albuquerque Lopes, Rênnis Oliveira da Silva, Yuri Wanderley Cavalcanti, Rafael Aiello Bomfim

**Affiliations:** I Universidade Federal da Paraíba, Programa de Pós-Graduação em Odontologia – João Pessoa (PB), Brasil.; II Universidade Federal da Paraíba, Centro de Ciências da Saúde da João Pessoa, Departamento de Odontologia Clínica e Social – João Pessoa (PB), Brasil.; III Universidade Federal de Mato Grosso do Sul, Faculdade de Odontologia, Departamento de Odontologia Social – Campo Grande (MS), Brasil.

**Keywords:** Saúde bucal, Desigualdades sociais, Idosos, Serviços públicos de saúde

## Abstract

**Objetivo:**

Analisar fatores individuais e contextuais associados ao uso de serviços públicos odontológicos entre idosos brasileiros, com base nos dados do SB Brasil 2023.

**Métodos:**

Estudo transversal com 9.745 idosos. O desfecho foi a utilização de serviços públicos de saúde bucal, em oposição ao uso de serviços privados. As análises consideraram o desenho amostral complexo e utilizaram regressão logística multinível com intercepto aleatório por município, orientada pelo Modelo Comportamental de Andersen. As variáveis incluíram fatores predisponentes (raça/cor, sexo e escolaridade), facilitadores individuais e contextuais (renda *per capita* e cobertura de saúde bucal na Atenção Primária à Saúde) e fatores de necessidade (edentulismo, necessidade percebida de tratamento dentário e de prótese).

**Resultados:**

A prevalência ponderada do uso de serviços públicos odontológicos foi de 39,2% (intervalo de confiança de 95% — IC95% 35,7–42,9). Após ajuste, observou-se maior chance de utilização do Sistema Único de Saúde (SUS) entre idosos pretos (*odds ratio* — OR 1,97; IC95% 1,60–2,40), pardos (OR 1,38; IC95% 1,19–1,61) e indígenas (OR 5,16; IC95% 2,01–13,21), bem como entre aqueles com edentulismo (OR 1,44), necessidade percebida de tratamento dentário (OR 1,26) e necessidade de prótese (OR 1,39). Residir em municípios com cobertura de saúde bucal superior a 70% associou-se a maior utilização do SUS (OR 2,47; IC95% 1,83–3,31). A variância atribuível ao nível municipal reduziu-se de 28,3% no modelo nulo para 16,7% no modelo final.

**Conclusão:**

A utilização de serviços públicos odontológicos entre idosos é socialmente estratificada e fortemente associada a vulnerabilidades sociais e necessidades em saúde bucal, evidenciando o papel pró-equidade do SUS.

## INTRODUÇÃO

O uso de serviços públicos de saúde bucal no Brasil é atravessado por importantes desigualdades sociais, raciais, de gênero e territoriais^1^. A população idosa, grupo prioritário quanto à necessidade de reabilitação oral, vivencia essas assimetrias em um contexto de maior vulnerabilidade econômica e social^2,3^. Evidências atestam que fatores como menor renda, baixa escolaridade e edentulismo estão associados a uma maior utilização dos serviços públicos de saúde bucal, refletindo não apenas padrões de iniquidade no acesso, mas também o papel estratégico do Sistema Único de Saúde (SUS) na ampliação da cobertura e no potencial de mitigação das desigualdades em saúde bucal^4,5^.

O Modelo Comportamental de Andersen constitui um referencial teórico amplamente consolidado para a análise da utilização de serviços de saúde, ao postular que o uso dos serviços resulta da interação entre fatores predisponentes, facilitadores e de necessidade, operando em níveis individual e contextual. Neste estudo, os fatores predisponentes individuais compreenderam características sociodemográficas como idade, sexo, raça/cor e escolaridade. Os fatores facilitadores incluíram atributos individuais, com destaque para a renda *per capita*, bem como características contextuais do município de residência, tais como a cobertura de saúde bucal na Atenção Primária à Saúde (APS) e a composição racial municipal. Os fatores de necessidade foram avaliados exclusivamente em nível individual, considerando-se tanto necessidades clínicas quanto percebidas, incluindo edentulismo, autopercepção da necessidade de tratamento odontológico, autopercepção da necessidade de prótese e motivo da utilização do serviço (tratamento, prevenção ou colocação/manutenção de prótese). Essa estrutura analítica permite examinar de forma sistemática como determinantes individuais e contextuais se articulam para moldar os padrões de utilização dos serviços públicos de saúde bucal em um sistema universal de saúde^6^.

No contexto brasileiro, torna-se fundamental incorporar a dimensão municipal na análise da utilização de serviços de saúde bucal, considerando-se o princípio da territorialização da APS e seu papel estruturante na organização do cuidado. A distribuição e a cobertura local da APS não apenas condicionam a oferta e o acesso aos serviços odontológicos, mas também interagem com marcadores sociais como raça/cor, renda, idade e gênero, produzindo padrões diferenciados de uso entre grupos populacionais^1,7,8^. Essa perspectiva é particularmente relevante em um sistema universal, no qual a equidade no acesso deve ser entendida como resultado da articulação entre características individuais e a capacidade organizacional dos serviços em nível local.

A literatura explica que a equidade no acesso e na utilização de serviços públicos de saúde não se restringe aos atributos individuais dos usuários, estando intrinsecamente relacionada à forma como os sistemas de saúde são organizados, financiados e implementados nos territórios^8^. Apesar disso, estudos que adotam abordagens multiníveis para analisar o uso de serviços odontológicos por idosos, incorporando simultaneamente variáveis contextuais do território, a composição demográfica e racial municipal e indicadores de necessidade em saúde bucal, ainda são escassos^9,10^. Ademais, mesmo entre investigações que utilizam modelos multiníveis, observa-se que a maioria não explora de forma sistemática variáveis relacionadas à raça/cor, à autopercepção da necessidade de prótese e aos motivos da utilização dos serviços, limitando a compreensão das desigualdades interseccionais no acesso aos cuidados odontológicos^11^.

Diante desse cenário, o presente estudo teve como objetivo analisar os fatores individuais e contextuais associados à utilização de serviços públicos de saúde bucal entre idosos brasileiros, com base nos dados da Pesquisa Nacional de Saúde Bucal — SB Brasil 2023, à luz do modelo comportamental de Andersen, contribuindo para o debate sobre equidade, organização territorial da APS e redução das desigualdades em saúde bucal no âmbito do SUS.

## MÉTODOS

Trata-se de um estudo transversal, de base populacional, que utilizou dados do inquérito nacional SB Brasil 2023 — Pesquisa Nacional de Saúde Bucal, coordenado pelo Ministério da Saúde. A pesquisa foi conduzida entre os anos de 2022 e 2023 e seguiu diretrizes metodológicas padronizadas em todo o território nacional, sendo o desenho do estudo descrito por Vargas et al.^12^ e Ferreira et al.^13^

O desenho amostral foi probabilístico e estratificado, garantindo representatividade para as capitais dos 26 estados, o Distrito Federal e áreas urbanas dos municípios do interior. A seleção da amostra foi realizada em diferentes etapas, com listagem prévia e sorteio sistemático de domicílios, além do uso de dispositivos eletrônicos e sistemas específicos de coleta e validação de dados em tempo real.

A coleta envolveu entrevistas domiciliares e exames clínicos padronizados, contemplando variáveis demográficas, socioeconômicas, autopercepção de saúde bucal, necessidade autorreferida de tratamento e uso de serviços odontológicos. A coleta de dados foi realizada por cirurgiões-dentistas e anotadores previamente treinados e calibrados utilizando instrumentos padronizados, como questionários estruturados e exames clínicos bucais. A padronização e a qualidade dos dados foram garantidas por meio de testes piloto, supervisões regulares e sistema eletrônico de coleta com validação em tempo real^12,13^.

No presente estudo, foram analisados 9.745 idosos com 60 anos ou mais que participaram do inquérito e que apresentavam informações completas para as variáveis de interesse. O desfecho principal foi o uso de serviços públicos de saúde bucal, identificado com base na pergunta sobre o local da última consulta odontológica. As respostas foram dicotomizadas entre atendimento pelo SUS e outros tipos de serviço (privado, conveniado ou outros).

Para analisar os fatores associados à utilização de serviços públicos de saúde bucal no SUS, as análises foram orientadas pelo modelo comportamental de Andersen e Newman^6^, que postula que o uso de serviços de saúde resulta da interação entre fatores predisponentes, facilitadores e de necessidade, operando em níveis individual e contextual. Considerando-se a estrutura hierárquica dos dados do SB Brasil 2023, com indivíduos aninhados em municípios, foi utilizada regressão logística multinível com intercepto aleatório por município, permitindo estimar simultaneamente os efeitos de variáveis individuais e contextuais, bem como a variância atribuível ao nível municipal.

As variáveis independentes foram organizadas conforme os componentes do modelo teórico. Os fatores predisponentes individuais incluíram sexo, raça/cor autodeclarada e escolaridade. Os fatores facilitadores abrangeram atributos individuais, como a renda *per capita*, e características contextuais do município de residência, incluindo a cobertura de saúde bucal na APS e a proporção de população negra no município. Os fatores de necessidade foram avaliados exclusivamente em nível individual e contemplaram tanto necessidades clínicas quanto percebidas, incluindo edentulismo, autopercepção da necessidade de tratamento odontológico, autopercepção da necessidade de prótese dentária, avaliação da saúde bucal e motivo da utilização do serviço.

Foram ajustados modelos hierárquicos sequenciais, iniciando-se por um modelo nulo, seguido por modelos com inclusão progressiva das variáveis individuais e contextuais, de acordo com o arcabouço teórico de Andersen. As análises foram realizadas no *software* Stata, versão 14.2 (StataCorp, EUA, Tx), considerando-se os pesos amostrais e o efeito do desenho amostral complexo do SB Brasil 2023. Os resultados foram expressos em *odds ratios* (OR) e intervalos de confiança de 95% (IC95%).

A Figura 1 ilustra o modelo conceitual de Andersen para explicar o uso de serviços públicos de saúde bucal por idosos.

**Figura 1:** Quadro conceitual do Modelo Comportamental de Andersen para a utilização dos serviços públicos de saúde bucal no Brasil (idosos).

O projeto SB Brasil 2023 foi conduzido em conformidade com a Resolução n^o^ 466/2012 do Conselho Nacional de Saúde e aprovado pela Comissão Nacional de Ética em Pesquisa (CONEP) sob Certificado de Apresentação para Apreciação Ética (CAAE) n^o^ 34497120.6.3001.0008. Todos os participantes, ou seus responsáveis legais, assinaram o Termo de Consentimento Livre e Esclarecido, e, para menores de 18 anos, foi obtido também o Termo de Assentimento Livre e Esclarecido.

### Declaração de disponibilidade de dados

Todo o conjunto de dados que dá suporte aos resultados deste estudo está disponível mediante solicitação ao autor.

## RESULTADOS

O estudo incluiu 9.745 idosos participantes da Pesquisa Nacional de Saúde Bucal — SB Brasil 2023. A Tabela 1 apresenta as características descritivas da população estudada, enquanto a Tabela 2 mostra os resultados da regressão logística multinível, organizados de acordo com os componentes do modelo comportamental de Andersen.

**Tabela 1:** Características descritivas e proporções. Estudo SB Brasil 2023 (n=9.745).

Variáveis individuais			n	% (proporção ponderada)	Uso público
					% (95%CI)
Prevalência					39,2% (35,7–42,9)
Grupo étnico					
	Brancos		3.601	49,5 (45,8–53,3)	32,0 (27,7–36,7)
	Pardos		4.465	36,1 (32,4–40,2)	44,5 (38,5–50,5)
	Amarelos		129	1,1 (0,7–1,7)	39,7 (25,3–56,1)
	Negros		1.353	12,8 (11,2–14,7)	52,9 (46,2–59,4)
	Indígenas		50	0,4 (0,2–1,0)	76,4 (51,2–90,9)
	Ignorado/sem informação		147	1,2 (0,7–2,0)	37,0 (19,3–59,1)
Sexo					
	Feminino		6.034	60,4 (58,2–62,7)	39,1 (35,7–42,7)
	Masculino		3.710	39,6 (37,3–41,9)	39,4 (33,8–45,2)
Renda *per capita* (salário-mínimo)					
	Até a linha de pobreza		621	6,7 (5,3–8,4)	56,6 (45,2–67,4)
	Pobreza até 1		2.263	27,6 (24,5–30,9)	51,9 (45,2–58,6)
	De 1 a 2		2.891	44,4 (40,7–48,3)	36,5 (31,1–42,3)
	Acima de 2		1.409	21,2 (18,0–24,9)	20,5 (13,2–30,5)
	Ignorado/sem informação		2.561	27,7 (23,4–32,5)	41,9 (35,2–48,8)
Escolaridade (anos)					
	Analfabeto		1.226	11,4 (9,8–13,3)	63,4 (55,8–70,4)
	De 1 a 4		2.485	58,7 (55,4–61,9)	42,3 (37,2–47,6)
	De 5 a 8		2.301	20,7 (18,2–23,5)	30,4 (25,1–36,2)
	Acima de 8		998	9,2 (7,4–11,3)	12,0 (8,4–16,9)
	Ignorado/sem informação		235	3,8 (2,4–5,7)	50,6 (37,6–63,5)
Contextual					
	Cobertura saúde bucal na Atenção Básica				
		Até 70%	7.300	72,3 (67,0–77,0)	34,0 (29,8–38,6)
		>70%	2.445	27,7 (22,9–33,0)	53,1 (47,6–58,5)
Proporção racial municipal					
	Até 60% de negros		4.494	65,8 (61,4–70,0)	33,0 (28,5–37,9)
	>60% de negros		5.291	34,2 (30,0–38,6)	51,9 (47,3–56,4)
Edêntulos					
	Não		6.230	63,5 (60,4–66,6)	38,1 (32,7–42,7)
	Sim		3.490	36,5 (33,4–39,6)	41,3 (36,6–46,2)
Autopercepção da necessidade de tratamento dentário					
	Não		4.434	49,0 (45,7–52,2)	32,1 (28,1–36,4)
	Sim		5.038	51,0 (47,8–54,3)	46,1 (41,6–50,6)
Autopercepção da necessidade de prótese					
	Não		3.793	42,6 (39,8–45,5)	33,5 (29,1–38,2)
	Sim		5.543	57,4 (54,5–60,2)	42,3 (37,3–47,5)
Motivo					
	Tratamento		4.092	45,5 (42,2–48,9)	46,9 (41,5–52,4)
	Prevenção		1.396	17,1 (14,4–20,2)	38,1 (29,8–47,2)
	Colocação/manutenção prótese		2.955	37,4 (22,9–41,1)	28,8 (24,1–34,1)

95%CI: intervalo de confiança de 95%.

**Tabela 2:** Fatores associados ao uso de serviço público odontológico (regressão logística multinível).

Variáveis			Uso de serviços públicos (OR)	
			Não ajustado	Ajustado*
			OR (95%CI)	OR (95%CI)
Fatores individuais predisponentes				
Raça/cor da pele				
	Brancos		1	1
	Preto		2,18 (0,84–1,29)	1.97 (1.60–2.40)
	Amarelo		1,47 (0,98–2,20)	1.53 (0.84–2.81)
	Pardos		1,66 (1,48–1,85)	1.38 (1.19–1.61)
	Indígenas		4,79 (2,36–9,72)	5.16 (2.01–13.21)
Sexo				
	Masculino		1	1
	Feminino		0,94 (0,83–1,02)	–
Escolaridade (anos estudo)				
	0		1	1
	1 a 4		0,66 (0,55–0,78)	0.65 (0.51–0.81)
	5 a 8		0,63 (0,53–0,75)	0.72 (0.58–0.91)
	>8		0,30 (0,26–0,36)	0.37 (0.30–0.47)
Fatores individuais facilitadores				
Per capita renda (equivalente) (salário-mínimo)				
	Até linha pobreza		1	1
	Linha pobreza a 1		0,71 (0,57–0,88)	0.84 (0.66–1.07)
	1 a 2		0,50 (0,41–0,62)	0.67 (0.53–0.86)
	Acima 2		0,16 (0,12–0,20)	0.26 (0.20–0.35)
Fatores facilitadores contextuais				
Cobertura SB-AB				
Até 70%			1	1
Acima 70%			2,83 (2,13–3,74)	2.47 (1.83–3.31)
Proporção racial				
	Até 60% negros			
	Acima 60% negros		2,38(1,77–3,19)	1.25 (0.93–1.69)
Fatores necessidades individuais				
Edentulismo				
	Não			
	Sim		0,84 (0,75–0,92)	1.44 (1.22–1.70)
Necessidade tratamento dentário				
	Não			
	Sim		1,80 (1,63–1,99)	1.26 (1.08–1.46)
Autoavaliação necessidade prótese				
	Não			
	Sim		1,75(1,59–1,93)	1.39 (1.21–1.59)
Motivo consulta				
	Prevenção		1	
	Tratamento		1,68(1,46–1,92)	1,09 (0,92–1,30)
Manutenção/colocação prótese			0,38 (0,33–0,44)	0,19 (0,16–0,24)
	AIC	modelo nulo	11.198	6.575
	BIC	modelo nulo	11.212	6.702
	VPC	modelo nulo	28,3% (22,7–34,7)	16,7% (11,4–23,7)

OR: odds ratio; 95%CI: intervalo de confiança de 95%; AIC: Akaike information criteria; BIC: Bayesian information criteria; VPC: Coeficiente de variação percentual (% de variação no nível das cidades).

### Fatores predisponentes individuais

No bloco dos fatores predisponentes, a raça/cor da pele manteve associação significativa com a utilização dos serviços públicos odontológicos após o ajuste por variáveis individuais e contextuais. Em comparação aos idosos brancos, observou-se maior chance de uso do SUS entre idosos pretos (OR 1,97; IC95% 1,60–2,40), pardos (OR 1,38; IC95% 1,19–1,61) e indígenas (OR 5,16; IC95% 2,01–13,21). A categoria amarela não apresentou associação estatisticamente significativa no modelo ajustado.

O sexo não se associou de forma independente à utilização dos serviços públicos odontológicos. Com relação à escolaridade, verificou-se um gradiente inverso: quanto maior o nível educacional, menor a chance de uso do SUS. Idosos com mais de oito anos de estudo apresentaram redução expressiva na chance de utilização (OR 0,37; IC95% 0,30–0,47) quando comparados aos analfabetos.

### Fatores facilitadores individuais e contextuais

Entre os fatores facilitadores individuais, a renda *per capita* mostrou associação inversa com a utilização dos serviços públicos. Idosos com renda entre um e dois salários-mínimos apresentaram menor chance de uso do SUS (OR 0,67; IC95% 0,53–0,86), e aqueles com renda acima de dois salários-mínimos apresentaram 74% menos chance de utilização (OR 0,26; IC95% 0,20–0,35) em relação aos idosos em situação de pobreza.

No nível contextual, a cobertura de saúde bucal na Atenção Básica apresentou associação robusta com o desfecho. Idosos residentes em municípios com cobertura superior a 70% apresentaram mais que o dobro de chance de utilizar os serviços públicos odontológicos (OR 2,47; IC95% 1,83–3,31) em comparação àqueles que viviam em municípios com menor cobertura. Por outro lado, a proporção racial municipal não manteve associação estatisticamente significativa após o ajuste (OR 1,25; IC95% 0,93–1,69).

### Fatores de necessidade individuais

No bloco dos fatores de necessidade, o edentulismo mostrou associação positiva com a utilização dos serviços públicos odontológicos no modelo ajustado (OR 1,44; IC95% 1,22–1,70). Da mesma forma, idosos que relataram necessidade de tratamento dentário (OR 1,26; IC95% 1,08–1,46) e necessidade de prótese dentária (OR 1,39; IC95% 1,21–1,59) apresentaram maiores chances de uso do SUS.

Quanto ao motivo da consulta, a busca por manutenção ou colocação de prótese esteve associada a menor chance de utilização dos serviços públicos (OR 0,19; IC95% 0,16–0,24), quando comparada às consultas motivadas por ações preventivas. O motivo do tratamento não manteve associação estatisticamente significativa após o ajuste.

#### Variância entre municípios

A análise multinível demonstrou que, no modelo nulo, 28,3% da variabilidade na utilização dos serviços públicos odontológicos era atribuível às diferenças entre os municípios. Após a inclusão das variáveis individuais e contextuais, esse percentual foi reduzido para 16,7%, demonstrando que parte substancial da variação no uso do SUS é explicada pelos fatores contemplados no modelo comportamental de Andersen.

## DISCUSSÃO

O presente estudo, com base nos dados do SB Brasil 2023, analisou a utilização de serviços públicos odontológicos à luz do modelo comportamental de Andersen, segundo o qual o uso dos serviços resulta da interação entre fatores predisponentes, facilitadores e de necessidade, operando em níveis individual e contextual. No componente dos fatores predisponentes, observou-se maior probabilidade de uso do SUS entre idosos autodeclarados pretos, pardos e indígenas, independentemente de outras características individuais e contextuais. No âmbito dos fatores facilitadores, destacaram-se a menor renda *per capita* e a menor escolaridade como determinantes centrais da maior dependência dos serviços públicos odontológicos, revelando um gradiente socioeconômico inverso no uso do SUS. Por sua vez, os fatores de necessidade, representados pelo edentulismo e pela percepção da necessidade de tratamento ou de prótese dentária, mostraram-se fortemente associados à utilização dos serviços, mostrando que o SUS atua prioritariamente no atendimento das demandas acumuladas e percebidas em saúde bucal entre idosos em situação de maior vulnerabilidade social e clínica.

Além disso, observou-se menor probabilidade de uso dos serviços públicos entre idosos que buscaram atendimento por motivos preventivos (como consultas de rotina e ações educativas) ou reabilitadores (por exemplo, confecção e manutenção de próteses dentárias), quando comparados àqueles que procuraram serviços por necessidade de tratamento curativo, como extrações, restaurações ou controle de dor. No nível contextual, municípios com maior cobertura populacional por equipes de saúde bucal (ESB) na APS e com maior proporção de população negra apresentaram maior chance de utilização dos serviços públicos.

Uma revisão de escopo conduzida por Ghanbari-Jahromi et al.^14^ identificou diversos fatores que influenciam o uso de serviços odontológicos entre idosos, destacando a renda, escolaridade, percepção de necessidade, edentulismo e barreiras contextuais como disponibilidade e custo. Esses achados são coerentes com os resultados do presente estudo, que também ressaltaram que fatores socioeconômicos e percepção de necessidade estão relacionados ao uso preferencial do SUS.

O modelo comportamental de Andersen, proposto em 1968 e revisado ao longo das décadas^15^, é um dos modelos teóricos empregados para compreender a utilização dos serviços de saúde. Segundo esse modelo, o uso dos serviços é influenciado por três dimensões: fatores predisponentes (como idade, escolaridade e raça/cor), fatores facilitadores (como renda e disponibilidade de serviços) e fatores de necessidade (como percepção do estado de saúde ou presença de condições clínicas). Neste estudo, foi identificada maior chance de utilização dos serviços públicos entre idosos que buscaram atendimento por motivos curativos, o que reforça a relevância dos fatores de necessidade como principal motivo para a procura por cuidados odontológicos. Também se verificou que municípios com maior cobertura por ESB apresentaram maiores chances de uso desses serviços, indicando a presença como fator facilitador relacionado à disponibilidade da rede. A maior prevalência de uso dos serviços públicos entre pessoas negras pode ser compreendida como um fator predisponente no modelo de Andersen, visto que essa população apresenta, historicamente, maior dependência do SUS. Em estudo, Constante identificou que pardos apresentaram 1,25 vez e negros 1,73 vez mais chance de recorrer aos serviços odontológicos públicos comparativamente aos brancos^16^.

Os achados deste estudo corroboram Martins et al.^17^, que, ao analisarem dados do SB Brasil 2010, identificaram maior uso dos serviços públicos por idosos com baixa renda, menor escolaridade, pretos ou pardos e com autopercepção negativa da saúde bucal. A presença de ESB no município também esteve associada ao uso, destacando a influência de fatores facilitadores e contextuais na ampliação do acesso. Os achados repetem o padrão que foi encontrado no SB 2010.

Ainda, Moreira et al.^18^, por meio de análise de classes latentes, também sublinharam que o grupo "perfil SUS" reunia idosos com piores condições socioeconômicas, necessidade de prótese e percepção negativa da saúde bucal, características semelhantes às encontradas em nosso estudo. Faz-se necessário apontar que, ao longo de 13 anos, o perfil das pessoas que acessam o SUS não modificou significativamente. Em certo sentido, isso revela que o setor público de saúde acolhe as pessoas que mais necessitam. Por outro lado, esse achado é alarmante, dado o montante investido na saúde bucal do SUS. Considerando-se a população idosa, é possível que as melhorias decorrentes da implementação e expansão da saúde bucal no SUS não tenham sido suficientes para produzir mudanças nas condições bucais desses indivíduos, especialmente em razão do acúmulo de problemas bucais desses indivíduos em momento anterior à Política Nacional de Saúde Bucal (PNSB).

Dados mais recentes do SB Brasil 2023 analisados por Pinto et al.^19^ reafirmam esse padrão, com maior uso do SUS entre adultos de 35 a 44 anos com baixa escolaridade, baixa renda, pretos ou pardos, sem plano de saúde, com edentulismo total, pior autopercepção da saúde bucal e necessidade percebida de tratamento. Embora o estudo tenha enfocado adultos, os padrões observados coincidem com os encontrados entre os idosos neste estudo, ressaltando a persistência das desigualdades no acesso. Ainda que a PNSB tenha ampliado o acesso aos serviços odontológicos no âmbito do SUS e contribuído para a redução de desigualdades raciais e sociais em saúde bucal^20^, os achados deste estudo demonstram que a utilização dos serviços públicos permanece concentrada entre grupos socialmente mais vulneráveis. Longe de representar uma limitação do SUS, esse padrão reforça seu papel como mecanismo pró-equidade, ao garantir acesso prioritário àqueles com maior carga de necessidades acumuladas em saúde bucal. A persistência desse perfil de usuários ao longo do tempo deve ser interpretada à luz das iniquidades estruturais históricas, marcadas pelo acesso tardio ou insuficiente aos serviços odontológicos ao longo do curso de vida, especialmente entre a população idosa. Nesse sentido, a elevada demanda observada entre idosos mais vulneráveis reflete, em grande medida, o acúmulo de agravos bucais anteriores à implementação da PNSB, bem como a natureza crônica e cumulativa das condições de saúde bucal, e não uma insuficiência das políticas públicas atuais em modificar, no curto prazo, desfechos fortemente determinados por trajetórias sociais passadas.

Embora os achados sugiram maior uso dos serviços públicos entre idosos edêntulos, a regressão indicou menor chance de utilização, sugerindo que o edentulismo pode atuar como barreira ao acesso, especialmente entre os mais vulneráveis. A perda total ou parcial dos dentes, associada a outras desigualdades, pode gerar obstáculos que inviabilizam a utilização dos serviços públicos de saúde bucal. Uma possível explicação é a naturalização da perda dentária como parte do envelhecimento, sobretudo entre idosos com menor escolaridade. Na ausência de dor ou desconforto funcional evidente, a busca por tratamento, como a reabilitação protética, tende a ser negligenciada. Esse comportamento pode ser interpretado à luz do modelo de Andersen^21^, que reconhece a percepção subjetiva de necessidade como determinante central do uso de serviços.

Estudos com dados do SB Brasil 2010 identificaram alta necessidade de prótese entre idosos com baixa escolaridade, renda e pior autopercepção, salientando iniquidades no acesso à reabilitação^22,23^. Ribeiro et al.^24^ afirmam que a necessidade de prótese nem sempre se converte em busca por atendimento por conta da combinação de fatores individuais e contextuais, como renda insuficiente, baixa escolaridade, mobilidade reduzida e insuficiência de serviços públicos. Esse cenário sugere que, para muitos idosos, a reabilitação protética deixa de ser prioridade diante de outras limitações cotidianas. Ademais, há indícios de maior busca por reabilitação em serviços privados, possivelmente em razão de maior agilidade ou qualidade percebida. Nascimento et al. observaram que idosos usuários de prótese total tinham maior chance de atendimento em serviços privados^25^.

Fagundes et al., com dados da PNS 2019, mostraram maior uso de serviços odontológicos entre pessoas com maior renda e escolaridade, indicando predomínio do acesso privado, enquanto entre idosos a prevalência foi menor, evidenciando barreiras persistentes para grupos mais vulneráveis^26^. No cenário internacional, Cossio-Alva et al. identificaram que a percepção de necessidade foi o principal fator associado ao uso de serviços entre idosos peruanos, reforçando o papel dos determinantes sociais^27^.

Neste estudo, os fatores de necessidade mostraram forte associação com o uso dos serviços públicos, em interação com fatores predisponentes e facilitadores. A menor chance de uso entre idosos edêntulos, mesmo diante da necessidade, reforça a existência de barreiras ao cuidado reabilitador. Apesar das limitações inerentes ao delineamento transversal e ao uso de variáveis autorrelatadas, os achados são relevantes para orientar políticas públicas voltadas à ampliação do acesso reabilitador e à desnaturalização do edentulismo na velhice. A utilização de regressão logística multinível permitiu considerar fatores individuais e contextuais, ampliando a compreensão dos determinantes do uso dos serviços odontológicos públicos por idosos.
